# A lumpy bumpy stomach: The more the murkier

**DOI:** 10.4102/sajr.v26i1.2437

**Published:** 2022-06-28

**Authors:** Binit Sureka, Siddhi Chawla, Sudeep Khera, Ashish Agarwal, Chhagan L. Birda, Sandeep Bairwa

**Affiliations:** 1 Department of Radiology, All India Institute of Medical Sciences Jodhpur, Jodhpur, India; 2 Department of Pathology, All India Institute of Medical Sciences Jodhpur, Jodhpur, India; 3 Department of Gastroenterology, All India Institute of Medical Sciences Jodhpur, Jodhpur, India; 4 Department of Medical Oncology, All India Institute of Medical Sciences Jodhpur, Jodhpur, India

**Keywords:** primary gastric lymphoma, DLBCL, MALT lymphoma, stomach thickening, gastric wall thickening, computed tomography

## Abstract

This report describes the radiological and endoscopic findings in a 54-year-old male who presented with epigastric pain. The patient underwent an upper gastrointestinal (GI) barium study followed by axial imaging, which demonstrated nodular gastric wall thickening. The classic findings of aggressive primary gastric diffuse large B-Cell lymphoma are presented with a brief review differentiating the pathological subtypes, important for patient prognostication and planning of therapy.

## Introduction

Primary gastric lymphomas are rare, however, the stomach is the most common gastrointestinal (GI) site of involvement in extra-nodal disease.^1^ Lymphomas may have diverse radiological manifestations, which can mimic a variety of benign diseases and malignant pathologies as enumerated in Box 1.^2^ Most of the lymphomas are either of the two most common types of B-cell non-Hodgkin lymphoma (NHL), that is, mucosa-associated lymphoid tissue (MALT) lymphoma and diffuse large B-cell lymphoma (DLBCL). The radiological differentiation between the two types is difficult but necessary because the prognosis and management differs accordingly.^3^ Various imaging modalities such as fluoroscopic upper GI barium imaging, ultrasonography, CT and MRI are used for the pre-treatment assessment and staging of upper GI malignant pathologies. This report describes the classic imaging findings of primary gastric lymphoma and possible differentials with a special emphasis on differentiating between the DLBCL and MALT subtypes.

BOX 1 Causes of gastric wall thickening.

**Table:** 

Etiology	Pathology
Gastritis/gastropathy	Menetrier’s disease Hypertrophic lymphocytic gastritis Hypertrophic hypersecretory gastropathy Acute gastritis with G-cell hyperplasia
Malignant causes	Diffuse gastric carcinoma Gastric adenocarcinoma Lymphoma
Polyps or polyposis syndromes	Juvenile polyposis Hyperplastic gastric polyps Hamartomatous polyps Cronkite-Canada syndrome Familial adenomatous polyposis
Infiltrative diseases	Amyloidosis Sarcoidosis
Miscellaneous causes	Proton-pump inhibitors Parietal cell hyperplasia Gastric antral vascular ectasia Zollinger-Ellison syndrome

*Source*: Agarwala R, Shah J, Dutta U. Thickened gastric folds: Approach. J Dig Endosc. 2018;09(04):149–154. https://doi.org/10.4103/jde.JDE_72_18

## Case presentation

A 54-year-old male patient presented to our hospital with symptoms of chronic epigastric pain accompanied by postprandial discomfort, early satiety and 2 kg of weight loss in the previous three months. There was no history of haematemesis, melena or vomiting. The liver and kidney function tests were within normal limits.

**FIGURE 1:**
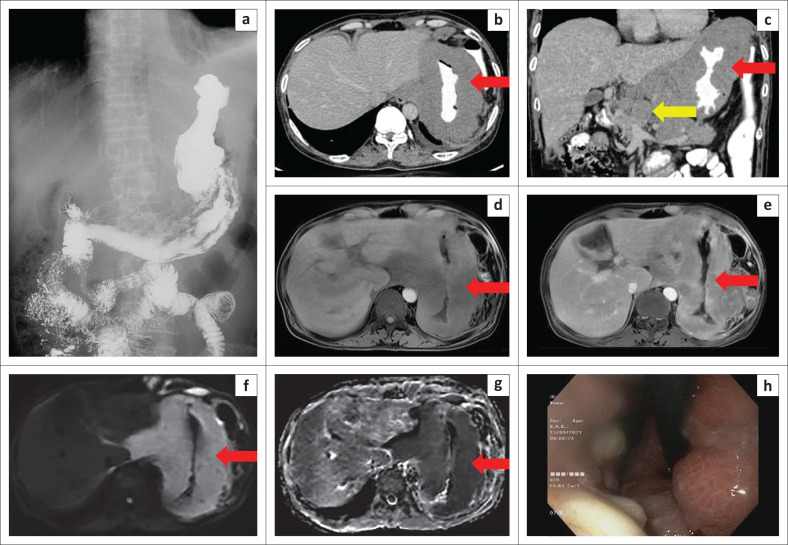
(a) Narrowing of the gastric lumen in the fundus and proximal body. (b, c) Axial and coronal contrast enhanced CT sections reveal significant gastric wall thickening with effacement of the mucosal folds (red arrows) and multiple enlarged discrete lymphnodes (yellow arrow). (d–g) Axial MRI pre- and post-contrast T1 weighted images, diffusion weighted image and ADC images demonstrate the gastric wall thickening with significant restriction (red arrows). (h) Upper GI endoscopy: Nodular thickening in the fundus of the stomach causing luminal stenosis.

As a result of the predominant upper GI complaints, the patient underwent an upper GI barium study, which demonstrated circumferential gastric luminal narrowing in the region of the fundus and body without obvious mucosal irregularity (Figure 1a). At a subsequent contrast enhanced CT scan, homogenous minimally enhancing circumferential thickening of the gastric wall (measuring ~2.5 cm) in the region of fundus and body was seen (Figure 1b, c) with associated multiple discrete similarly enhancing supra- and infra-diaphragmatic lymph nodes (Figure 1c). The MRI scan revealed isointense thickening on T1 weighted fat saturated images (Figure 1d), showing homogenous enhancement on post-contrast T1 weighed images (Figure 1e) in the same region with prominent diffusion restriction (Figure 1f, g). In view of the degree of thickening and associated lymphadenopathy, gastric lymphoma was presented as an imaging differential.

**FIGURE 2:**
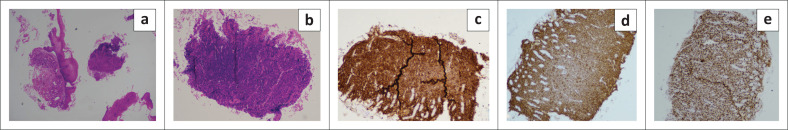
(a) Hematoxilin and eosin stain (H&E), 4× Fragmented gastric biopsy shows a cellular fragment, (b) H&E, 10×: Higher magnification of the cellular fragment comprising of high nuclear/cytoplasmic (N/C) ratio cells, (c) immunohistochemistry (IHC), CD45: Atypical lymphoid cells show diffuse membranous expression of CD45, (d) IHC, CD20: Atypical lymphoid cells show diffuse membranous expression of CD20, (e) IHC, Ki-67: High labelling index of Ki-67 expressed by the atypical lymphoid cells.

Upper GI endoscopy (Figure 1h) demonstrated a thickened and lobulated appearance of the fundus causing moderate luminal narrowing, however, no ulcerations or mucosal erosions were seen. Histology on the biopsy acquired reported sheets of atypical lymphoid cells in the subepithelium (Figure 2a), intermediate to large in size, exhibiting moderate nuclear pleomorphism, a high nuclear-cytoplasmic ratio, vesicular chromatin, conspicuous nucleoli and a scant amount of cytoplasm (Figure 2b). These cells were found to infiltrate the submucosal glands. On immunohistochemistry, the tumour cells were positive for CD45 (leucocyte common antigen [LCA]) (Figure 2c) and CD20 (Figure 2d) and negative for CK & CD3. Ki-67 labelling index was 90% – 95% (Figure 2e). These findings supported a primary high-grade gastric lymphoma – DLBCL type.

The patient was initiated on R-CHOP regimen (Rituximab, Cyclophosphamide, Doxorubicin, Oncovin [Vincristine], and Prednisone) chemotherapy.

## Discussion

Primary gastric lymphomas are diagnosed using the criteria by Dawson et al.^4^, which includes predominant involvement of the stomach along with nodal involvement confined to its drainage area, no other palpable superficial lymphnodes, a normal chest radiograph, normal total leukocyte counts and no involvement of the other organs such as the liver or spleen. Most GI lymphomas represent the non-Hodgkin’s subtype with the previous studies reporting Hodgkin’s lymphoma only in few cases.^5,6^ The incidence of NHL has been increasing over time because of various risk factors, which include HIV infection, *Helicobacter pylori* infection, coeliac disease, inflammatory bowel disease and immunosuppression after solid organ transplantation.^7^

Under normal circumstances gastric mucosa is devoid of lymphoid tissue, however, with chronic *H. pylori* infection there is reactive development of lymphoid tissue within the lamina propria. Mucosa-associated lymphoid tissue lymphoma is a low-grade lymphoma and more than 70% of cases are secondary to chronic *H. pylori* infection whilst DLBCL, in contrast, is an aggressive and high-grade lymphoma, which has a poor prognosis with high rates of recurrence.^3,8^ Conventional upper GI barium studies and upper GI endoscopy are limited as they only assess luminal characteristics. This limitation is overcome in upper GI endoscopy by accompanying endoscopic ultrasound, which can assess the extra-luminal characteristics of the lumen and also assist in acquiring targeted biopsies.

Multiplanar reconstruction CT can better assess the gastric wall thickness, mucosal enhancement, lymph node involvement and other organ involvement and thus has become the investigation of choice for assessing lesions of the stomach.^9^ MRI is used for evaluation of lesions that are difficult to characterise at multidetentor CT. Lymphomas demonstrate significant restriction on diffusion weighted images (DWI) and corresponding apparent diffusion coefficient (ADC) maps because of their hypercellular nature. Positron emission tomography with computed tomography (PET/CT) scan is helpful in disease staging and treatment response assessment due to its high sensitivity.

Gastric wall thickness > 1 cm favours the diagnosis of lymphoma.^10^ In both DLBCL and MALT lymphoma, the antrum and body are the most common areas of involvement, however, heterogeneity of contrast enhancement, extensive thickening, serosal involvement and extra-nodal disease is more common with DLBCL. Multifocal involvement of the stomach, nodular and ulcerative morphology of lesions, which can lead to subsequent gastric stenosis, are more common in DLBCL. Mucosa-associated lymphoid tissue lymphoma on the other hand has an infiltrative nature and thus luminal stenosis is uncommon. Differences between these two types of lymphoma are documented in Table 1.

**TABLE 1:** Important differentiating features between diffuse large B-cell lymphoma and mucosa-associated lymphoid tissue-lymphomas of stomach.

Histological grade	DLBCL – high-grade	MALT lymphoma – low-grade
Aetiology	Proliferation of atypical lymphoid cells; May transform from pre-existing MALT lymphoma	Secondary to *H. Pylori* infection (70% cases)
Morphological types	Nodular, ulcerative, Infiltrative	Usually infiltrative
Gastric lumen	More extensive thickening; Gastric stenosis more common	Less extensive thickening; Gastric stenosis rare
Gastric serosal involvement	More common	Less common
Lymphadenopathy	Enlarged perigastric nodes	Less common
Multifocality	Common	Uncommon
Management	Combination chemotherapy and radiotherapy	*H. Pylori* eradication therapy
Prognosis	Poor	Relatively good

*Source:* Data obtain from the Department of Diagnostic and Interventional Radiology, All India institute of Medical Sciences, Jodhpur, Rajasthan, India.

DLBCL, diffuse large B-cell lymphoma; MALT, mucosa-associated lymphoid tissue.

Differentiation of lymphomatous gastric wall thickening from other malignant conditions, like adenocarcinoma, is essential. Adenocarcinoma is more likely to infiltrate the gastric wall and infiltrate the adjacent structures, whilst preservation of the perigastric fat planes is more probable in lymphoma.^7^ The lumen of the stomach remains patent even with extensive lymphomatous infiltration of the wall whilst in cases of adenocarcinoma the patient can present with gastric outlet obstruction because of the constricting and scirrhous nature of the pathology.^11^ The linitus plastica appearance related to infiltration of the submucosa of the gastric wall in scirrhous adenocarcinoma leading to decreased capacity and rigidity of stomach wall can also be seen in non-Hodgkin gastric lymphoma, however, in these cases lymphomatous cells are seen within the submucosa on pathological evaluation.^12^ Transpyloric spread of disease is also more common in lymphoma than adenocarcinoma however the higher incidence of carcinoma decreases the specificity of this differentiation.^13^ Lymph nodes in cases of lymphomas are homogenously enhancing and bulky and often seen to extend below the level of the renal hilum whereas they are smaller, necrotic and localised to the local drainage site of the stomach in cases of adenocarcinoma.^14,15^

In suspected cases of lymphoma and in cases where a diagnostic dilemma persistsupper GI endoscopy investigation is warranted. It can delineate the extent of the pathology and guided biopsy from suspicious areas will allow a histopathological diagnosis. Immunohistochemistry stains can further differentiate the subtypes of lymphoma as was described in the presented case. Once the diagnosis is confirmed, treatment of MALT lymphoma consists of anti-*H. Pylori* medication, which is initiated as early as possible once the diagnosis is confirmed.^9,16^ With DLBCL a combination of chemotherapy and radiation therapy can result in complete remission in up to 90% of cases.^16^

## Conclusion

This case demonstrates the usefulness of multimodality imaging and describes the classic imaging findings in the DLBCL type of gastric lymphoma. It is quintessential for radiologists and clinicians to be aware of this entity, differentiating it from other types of lymphomas and other malignant pathologies so that appropriate treatment is not delayed.
